# Breakfast Size and Prevalence of Metabolic Syndrome in the European Prospective Investigation into Cancer and Nutrition (EPIC) Spanish Cohort

**DOI:** 10.3390/nu15030630

**Published:** 2023-01-26

**Authors:** Leila Lujan-Barroso, Lucia Iglesias, Raúl Zamora-Ros, Cristina Lasheras, María-José Sánchez, Natalia Cabrera-Castro, Josu Delfrad, Pilar Amiano, Esther Molina-Montes, Sandra Colorado-Yohar, Conchi Moreno-Iribas, Ane Dorronsoro, Miguel Rodríguez-Barranco, María Dolores Chirlaque, Amaia Aizpurua, Antonio Agudo, José Ramón Quirós, Paula Jakszyn

**Affiliations:** 1 Unit of Nutrition and Cancer, Cancer Epidemiology Research Program, Catalan Institute of Oncology—IDIBELL, L’Hospitalet de Llobregat, 08908 Barcelona, Spain; 2 Department of Public Health, Mental Health and Mother-Infant Nursing, School of Nursing, Faculty of Medicine and Health Sciences, University of Barcelona, 08907 Barcelona, Spain; 3 Department of Functional Biology, University of Oviedo, 33007 Oviedo, Spain; 4 Escuela Andaluza de Salud Pública (EASP), 18011 Granada, Spain; 5 Instituto de Investigación Biosanitaria ibs.GRANADA, 18012 Granada, Spain; 6 Centro de Investigación Biomédica en Red de Epidemiología y Salud Pública (CIBERESP), 28029 Madrid, Spain; 7 Department of Preventive Medicine and Public Health, University of Granada, 18071 Granada, Spain; 8 Department of Epidemiology, Murcia Regional Health Council, IMIB-Arrixaca, 30120 Murcia, Spain; 9 Navarra Public Health Institute, 31003 Pamplona, Spain; 10 Navarra Institute for Health Research (IdiSNA), 31008 Pamplona, Spain; 11 Sub-Directorate for Public Health and Addictions of Gipuzkoa, 20010 Donostia, Spain; 12 Biodonostia Health Research Institute, Epidemiology of Chronic and Communicable Diseases Group, 20014 San Sebastián, Spain; 13 Department of Nutrition and Food Science, Campus of Cartuja, University of Granada, 18071 Granada, Spain; 14 Institute of Nutrition and Food Technology (INYTA) ‘José Mataix’, Biomedical Research Centre, University of Granada, 18071 Granada, Spain; 15 Research Group on Demography and Health, National Faculty of Public Health, University of Antioquia, Medellín 050010, Colombia; 16 Sociohealth Sciences Department, Murcia University, 30100 Murcia, Spain; 17 Blanquerna Health Sciences Faculty, Ramon Llull University, 08022 Barcelona, Spain

**Keywords:** chrononutrition, breakfast, metabolic syndrome, meal timing

## Abstract

Background: Recent evidence suggest that energy distribution during the daytimecould be a potential determinant for the development of metabolic syndrome (MetS). Objective: To cross-sectionally assess the association between breakfast size and the prevalence of MetS in Spanish adults. Methods: Our study included a subset of 3644 participants from the European Prospective Investigation into Cancer and Nutrition Spain study recontacted between 2017–2018. Information on diet, sociodemographic, lifestyle, sleep quality, and chronotype was collected using standardized questionnaires, while anthropometric and blood pressure data were measured in a face-to-face personal interview by a nurse. MetS was defined according to the Adult Treatment Panel III (ATPIII) definition by measuring serum levels of total cholesterol, tryglycerides and glucose. Breakfast size was calculated as: (energy from breakfast/total energy intake) * 2000 kcal. To evaluate the association between breakfast size and MetS prevalence, a multivariable logistic regression model adjusted by potential confounders was used to estimate OR and 95% CI. Results: Prevalence of MetS in our study was 40.7%. The mean breakfast size was 306.6 * 2000 kcal (15% of the total daily energy intake), with 14 (0.4%) participants skipping breakfast. Participants in the highest quartile of breakfast size had a lower MetS prevalence compared to participants in the lowest quartile (OR_Q4vsQ1_ = 0.62; 95% CI = 0.51–0.76; *p*-trend < 0.001). No modification of the estimated ORs by sex, breakfast time, and number of eating occasions per day were observed. Conclusion: Our results suggest that higher breakfast size is associated with lower prevalence of MetS in Spanish adults, supporting the importance of a high energy breakfast. Further prospective studies are necessary to confirm these findings.

## 1. Introduction

Worldwide, prevalence and trends of metabolic syndrome (MetS) are rapidly increasing, along with prevalence of obesity, insulin resistance and type 2 diabetes [1]. In Spain, prevalence of Mets is around 40% and 32% in older men and women respectively [2] Although prevalence estimates vary based on the criteria used for defining MetS: WHO 1999 [3], the Adult Treatment Panel III (ATP-III) 2005 [4], and the International Diabetes Federation (IDF) 2006 [5], around a quarter of the worldwide adult population suffers from MetS according to the most updated reports [1,2,6]. 

Several factors, including genetic, sedentary lifestyle, and unhealthy diet, have been associated with the increasing prevalence of MetS [1]. Eating and sleeping behavioral patterns in modern living are misaligned with our body’s natural biological daily cycles and have been associated with many detrimental effects on health such as cardiovascular disorders, type 2 diabetes, obesity, and cancer [1]. In the last decades, the field of chronobiology has shed some light on the role that individual chronotype, meal timing, and energy distribution throughout the day could play as potential determinants for MetS [7]. 

Breakfast is often considered the most important meal [8] and many studies have widely assessed the harmful effect of breakfast skipping on health outcomes [9,10,11,12,13,14,15]. In addition, a recent Australian cross-sectional study including 9341 adults [16] suggested that obtaining a higher proportion of daily energy intake from breakfast may contribute to a better diet quality and lower total daily energy intake. 

Besides the size of breakfast, also its quality and macronutrients composition have been studied in recent years in relation to cardiometabolic health, glycemic control, and individual biomarkers of metabolism [17,18,19,20,21,22,23,24,25]. For example, high carbohydrate intake and lower amount of fat intake during breakfast were associated with a healthier cardiometabolic profile in adults [19,20,21]. Regarding meal timing and its impact on metabolism and weight control, available research has mainly assessed lunch, dinner and night eating, but not breakfast [10,21,22,23,24,25,26,27,28,29].

According to some reports addressing the question of what percentage of total energy intake should breakfast cover, the most common proposed range is between 15% and 25% of the daily energy [30]. Some mechanisms have been proposed as potential mediators of the effect of breakfast on weight, body mass index (BMI) and metabolic profile. These include lifestyle characteristics, such as increasing physical activity (PA) and healthier food choices, increasing satiety associated with morning intake, and meal-timing-dependent energy expenditure [8,31]. Thus, despite the increasing interest regarding the influence of energy distribution across meals on metabolic profile and obesity, the relation between breakfast size and MetS remains little investigated. So, our aim was to cross-sectionally assess the association between breakfast size and the prevalence of MetS in Spanish adults.

## 2. Materials and Methods

### 2.1. Study Design and Population

This study was carried out in subjects from the Spanish cohort of the European Prospective Investigation into Cancer and Nutrition (EPIC-Spain). The EPIC-Spain cohort included 41,437 subjects aged 29–69 years, who were recruited between 1992 and 1996 from five regions of Spain: Asturias, Granada, Murcia, Navarra, and Gipuzkoa. Further details about the EPIC-Spain cohort can be found elsewhere [32,33]. 

The subset of participants elegible for this study were those who, by December 2015, were alive and younger than 67 years (for males) and younger than 60 years (for females), a total of 8000 people. The age restriction was based on evidence showing that the effect of chronotype and social jetlag diminishes in older generations [1] while the different sex-specific cut-off points for ages allowed us to obtain a balanced sample by sex. In total, the initial sample included 4224 (75.5%) subjects, of which 2540 were women (892 from Asturias, 824 from Granada, 885 from Murcia, 795 from Navarra, and 828 from Gipuzkoa) (Supplementary Figure S1) [34].

Signed informed consent forms were retrieved from all participating individuals, and approval was granted to each center from their local Ethics Committees (54/U/2016).

### 2.2. Data Collection

The initial sample of this analysis (*n* = 4224) was recontacted via telephone, between 2017 and 2018, and was interviewed by trained staff to obtain information related to their diet (relevant for chrono-nutrition) and sleeping patterns (relevant for defining chronotype). During the telephone interview, data on sleep quality were also collected by means of the Pittsburgh Sleep Quality Index (PSQI) questionnaire [35]. Chronotype was assessed through information on sleeping habits, and based on data collected using the questions from both the Horne-Ostberg’s Morningness-Eveningness Questionnaire (MEQ) and the Munich Chrono-type Questionnaire (MCTQ) [36,37]. Individual chronotypes were classified as either moderate/extreme early, slight early, normal, slight late or moderate/extreme late types, according to the obtained scores after having set the cut-off points at percentiles 2.5, 10, 90 and 97.5.

In addition, sociodemographic and general lifestyle information (such as laboral physical activity, non-laboral physical activity–including leisure-time, housework, and vigorous activity, history of smoking, reproductive history, and some chronic diseases) were also collected. Finally, all participants were invited to a face-to-face personal visit with a nurse for the collection of anthropometric measurements (height, weight, waist circumference and body composition analysis), blood pressure, and a fasting blood sample. All measurments have followed standarized measurements as we have previously described [34]. A total of 4031 (95.4%) and 3772 (89.3%) subjects agreed to participate in the nurse interview and blood collection, respectively (Supplementary Figure S1).

### 2.3. Biological Samples

During the personal interview a 30 mL blood sample was taken from those participants who agreed to participate in the blood collection process (*n* = 3772). The samples were taken in a fasting (at least 12 h since the last intake) following standard protocols, and were stored at −80 °C. The serum samples were analyzed by standardized enzymatic laboratory techniques to obtain concentrations of specific biochemical parameters (i.e., glucose, triglycerides and cholesterol parameters (total, high-density lipoprotein [HDL], and low-density lipoprotein [LDL]).

### 2.4. Metabolic Syndrome Definition

We defined MetS according to the ATP-III [4] criteria. This criterion defines an individual as having MetS when three of the following five components are present: (i) waist circumference ≥ 102 cm in men and ≥88 cm in women; (ii) hypertriglyceridemia, triglycerides ≥ 150 mg/dL (1695 mmol/L); (iii) low HDL-cholesterol, <40 mg/dL (0.9 mmol/L) in men and <50 mg/dL (1.1 mmol/L) in women; (iv) hypertension, blood pressure ≥ 130/85 mmHg or hypertension reported as a chronic disease; and (v) hyperglycaemia, fasting glucose ≥ 100 mg/dL (≥6.1 mmol/L) or type 2 diabetes reported as a chronic disease.

### 2.5. Dietary Information

For this study, information on diet was collected using a validated diet history [34] structured by eating occasion and the specific time of day that foods were ingested. Food consumption was registered in grams/day. A total of 10 eating and time was considered: immediately after waking up, breakfast, mid-morning, lunch, afternoon, dinner, before sleep, before lunch, before dinner, during the night. In the case of lunch and dinner, were asked in relation: starter, first and second course, side dish, desserts, and beverages, and bread.

We considered breakfast as the first self-reported meal of the day, which included at least one food group. The consumption of only non-caloric beverages, such as coffee, tea, was considered as skipping breakfast [8]. We described the consumption of total and simple carbohydrates, fiber, proteins (including animal and plant-based proteins) and fats. In addition, the percent of energy from carbohydrates, proteins, and fats for the entire day and at breakfast, as well as the percent of energy at breakfast from the total, were computed. The Spanish Food composition database was used to calculate total energy, macronutrients from the individual food consumption data. Finally, breakfast contribution to total energy was calculated as follows: (energy from breakfast/total energy intake) * 2000 kcal.

### 2.6. Statistical Analysis

Medians and percentiles 25 (p25) and 75 (p75) were used for continuous variables. For categorical variables, bivariate analyses were performed by MetS status and by breakfast size categories, as absolute and relative frequencies. Chi-square test and Wilcoxon rank sum test were used for categorical and continuous variables, respectively overall and by components.

**Figure 1:**
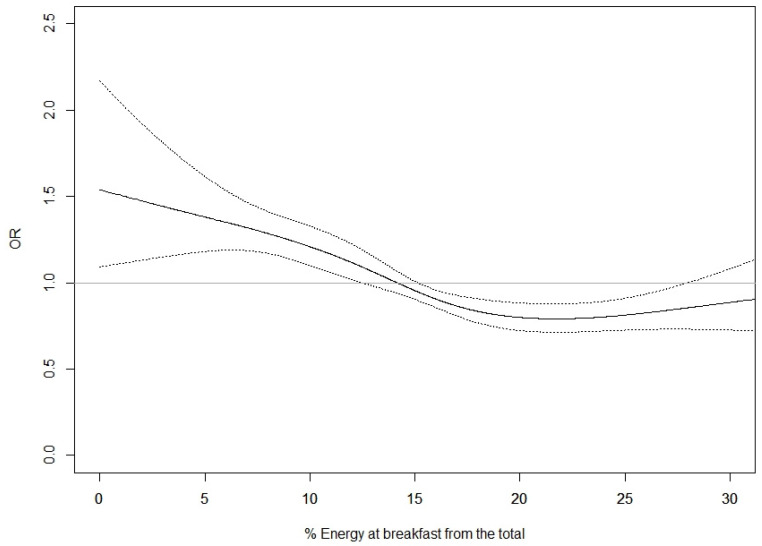
Percentage of energy at breakfast from the total daily energy intake and prevalence of Metabolic Syndrome. Logistic regression model was adjusted for center, sex, age, educational level, recreational physical activity, number of eating occasions (≤3, 4–5, >5), and breakfast time (≤9:00, >9:00).

To evaluate the association between breakfast size and MetS prevalence, a logistic regression model was used to estimate prevalence odds ratio (OR) and 95% confidence interval (95% CI). Breakfast size was evaluated using both the categorical variables based on quartiles. Furthermore, we evaluated the association between breakfast size and each of the five components used in the MetS definition. Restricted cubic splines (RCS) with 3–5 knots were used to explore linearity in the trend in the Mets prevalence with breakfast size. Since relationship was not lineal, continuous analysis was not presented. Akaike information criterion (AIC) was used to select the best representation of the relation between breakfast size and prevalence of MetS. The minimum AIC was found with a four-knot RCS (5, 35, 65, and 95th percentiles of the distribution of breakfast size) (Figure 1). 

Two models were built using logistic regression. The minimally adjusted model included sex, age (years), and center. The multivariate model was additionally adjusted for educational level (none, primary school, technical studies, secondary school, higher education, unknown), recreational Physical activity (MET-h/week), number of eating occasions per day (≤3, 4–5, >5), and breakfast time (≤9:00 h, >9:00 h). Smoking history, PA at home, vigorous PA, sleep quality, sleep duration, chronotype, time from wake up to first meal, lunch time, dinner time, and dietary intake at full day (including % energy from protein, % energy from carbohydrates, and % energy from fats) were not included as adjustment covariates because they were not statistically significant in the bivariate analysis. Modification of the estimated Ors by sex, breakfast time, and number of eating occasions per day was evaluated using a likelihood ratio test.

All statistical tests were two-sided and evaluated at a level of 0.05. All analyses were performed using SAS v 9.4. Restricted cubic splines analysis was performed using R v 3.6.3.

## 3. Results

### 3.1. Descriptive Statistics

A total of 3644 subjects (40.7% with MetS) with biochemical and diet data (125 (3.3%) and 3 (0.08%) of the participants were excluded due to no information on diet or MetS status were available respectively) (Supplementary Figure S1), with a median age of 65 (62–69) years, out of which 39.8% were men, were included in this analysis. The mean breakfast size was 306.6 * 2000 kcal (SD = 138.4), with 14 (0.4%) participants skipping breakfast, corresponding at a 15% of the total daily intake. 

Sociodemographic and dietary variables by MetS condition are presented in Table 1. The prevalence of MetS was higher in those participants with lower educational levels (at most, primary school), higher BMI, higher % body fat, and those that were less active. A higher prevalence of breakfast late (>9:00 h), and higher number of eating occasions. was also seen in participants with MetS. Finally, higher prevalence with the following at-breakfast dietary pattern: lower energy contribution from breakfast, lower percentage of energy from carbohydrates, and higher percentage of energy from protein were observed in participants with MetS. Further, more detailed, information on diet can be found in Supplementary Table S1.

Table 2 shows the sociodemographic and dietary variables by quartiles of breakfast size. Lower breakfast size was observed in women (Q1: 52.9% vs. Q4: 64.7%) compared with men (Q1: 47.1% vs. Q4: 35.4%), in those participants with lower educational level (at most, primary school; Q1: 57.2% vs. Q4: 51.5%) compared with higher educational level (Q1: 16.5% vs. Q4: 23.6%), and former smokers (Q1: 40.9% vs. Q4: 36.96%) compared with never smokers (Q1: 40.2% vs. Q4: 53.3%). Participants in the lower quartile of breakfast size had higher BMI and lower MET-h/week of non-laboral PA, specifically recreational PA. The % of subjects with Abdominal obesity, hyper triglyceridemia and hyperglucèmia levels were low the higher quartile of breakfast size. Moreover, % of subjects with eating occasions higher than five decrease across quartiles of breakfast size (Q4 46.7% vs. Q1 54.2%).

Finally, regarding dietary intake, a reduction in daily total energy intake across quartiles of breakfast size (Q1: 2494.1 Kcal vs. Q4: 2322.3 Kcal) was found. Specifically at breakfast, we observed a statistically significant descending gradient from the lowest to the highest quartile of breakfast size regarding the percentage of energy from proteins (Q1: 16.1% vs. Q4: 12.4%) and carbohydrates (Q1: 58.3% and Q4: 53.4%); and the opposite for fats (Q1: 23% and Q4: 33.4%). Further details on diet are shown in the Supplemental Table S2, where the amount of dietary intake at breakfast for total and simple carbohydrates, fiber, proteins (both animal and plant-based), fats (saturated, monounsaturated, and polyunsaturated fats) across quartiles of breakfast size all show a statistically significant difference. Contrary to what happened at breakfast, the dietary intake of fiber, protein (both animal and plant-based) and fat on the full day decreased, as quartiles of breakfast size increased.

### 3.2. Breakfast Size and Prevalence of Metabolic Syndrome

Prevalence of MetS was inversely associated with breakfast size. After adjusting for relevant confounders, we observed a statistically significant inverse association between breakfast size and the prevalence of MetS (OR_Q4vsQ1_ = 0.62; 95%CI = 0.51–0.76) (Table 3). The model with detailed information on eating occasions and breakfast size can be seen in Supplemental Table S3, where both late breakfast time (>9:00 h vs. ≤9:00 h) and higher number of eating occasions (>5 vs. 4–5) were positive associated with MetS prevalence, in opposite with those with eating ocasions < than 3. The relation between breakfast size and each of the individual components of MetS were also analyzed. We observed that breakfast size was inversely related with the prevalence of the following individual components of MetS: abdominal obesity (OR_Q4vsQ1_ = 0.59; 95% CI = 0.48–0.72), hypertension (OR_Q4vsQ1_ = 0.57; 95% CI = 0.37–0.88), and hyperglycemia (OR_Q4vsQ1_ = 0.56; 95% CI = 0.46–0.68). No statistically significant associations were observed with the remaining (triglycerides, and HDL-cholesterol) components of Met even a similar tendency reletd to other components has been found (Table 3).

No modification of the estimated ORs were observed, by sex, breakfast time, or number of eating occasions per day (data not shown).

Figure 1 shows the non-lineal association between the percentage of energy at breakfast from the total energy regarding the prevalence risk of MetS for the multivariate logistic regression. The association reflects decreased prevalence of MetS for increased percentages of energy at breakfast, with the highest prevalence of MetS in cases where the energy percentage at breakfast, relative to the total daily energy intake, is lower than 15%. A plateau shape below the unit is observed from 15% to 29% of breakfast size. Statistical significance is lost beyond 29%, due to the small number (<4% of the sample) of participants within that range.

## 4. Discussion

To our knowledge, this is the first multicenter study reporting on the influence of breakfast size over the prevalence of MetS. The results arising from this cross-sectional analysis of 3644 Spanish adults showed 38% lower MetS prevalence in those participants with higher breakfast sizes, compared to those with lower breakfast sizes More specifically, our results showed lower prevalence of MetS when between 15 and 30% of the daily energy intake was covered by breakfast.

Traditionally, studies on metabolic health and weight control have highlighted the effects of breakfast skipping [9,10,11,12,13,14,15]. In recent years, a new area of interest has emerged, which has placed focus on meal timing, daily energy distribution and macronutrient composition by eating occasions [9,17,18,19,20,21,22,23,24,25,26,27,28,29,38,39,40]. In general terms, it has been found that greater energy intake earlier in the day and a high quality composition of breakfast are beneficial for weight control and metabolic health [9,17,18,19,20,21,22,23,24,25,26,27,28,29,38,39,40]. These data are in concordance with our results, which showed lower prevalence of MetS in participants with higher breakfast sizes definitions. In relation to specific MetS components, differences in exposure assessments and different components of the MetS studied make it difficult to establish comparisons. In this sense, overall MetS prevalence in relation to breakfast size has been little investigated.

Recently, a small prospective study in Spain [41], which included 607 subjects (101 cases), observed percentages of energy intake at breakfast similar to ours (17% and 15%, respectively). However, while we observed a lower prevalence of MetS on those participants with higher break sizes, they did not observe an association between the percentage of energy at breakfast and the incidence of MetS. They did, however, find that higher percentages of energy intake at dinner were associated with higher incidence of MetS (OR: 2.57; 95% CI = 1.14–5.79).

In two cross-sectional studies performed by de Castro, it was suggested that low energy density intake (kcal/g) in the morning could reduce the total daily energy intake [42,43], De Castro, 2007 [31] additionally observed that the association of morning intake with reduced total intake was macronutrient specific. Our data further show that higher breakfast sizes reduce the total amount of daily energy intake (Q1 = 2494 vs. Q4 = 2322 kcal/d). Their findings showed that the higher the breakfast consumption of carbohydrates, proteins and fats, the lower their respective daily intakes were. In contrast, we only observed this inverse association regarding fat intake. Our results specifically showed that, for those in the highest quartile of breakfast size, the greatest energy contribution came from fats (Q_1_ = 23% vs. Q_4_ = 33%, *p* < 0.001), which seemed to lead to decreased fat consumption during the rest of the day (Q_1_ = 104 g/d vs. Q_4_ = 97 g/d, *p* < 0.001). A recent Australian cross-sectional study [16] on breakfast size and diet quality, found that a higher proportion of daily energy intake provided by breakfast improved diet quality and reduced total energy intake. This could have been thought to have an indirect impact on BMI but strikingly, they observed no statistically significant association with breakfast size.

We observed that the highest proportion of participants reporting more than five eating occasions per day and a higher daily energy intake were in the lowest quartile of energy intake from breakfast. Furthermore, we found that eating more than five meals per day increased the prevalence of MetS by 23%, probably due to frequent snacking. In regard to frequency of meals, previous studies have shown controversial results. As reflected in a 2021 literature review, including observational and intervention studies results have shown contrasting results. By one hand, some results showed that a higher number of eating occasions being associated with lower BMI, lower energy density, lower alcohol intake, and higher fruit intake. By the other hand, other authors found that a high number of eating occasions increased food stimuli, hunger and the desire to eat, while consequently also increasing BMI, abdominal fat, and the risk of developing type 2 diabetes [44]. Our results support that the distribution of energy and macronutrients during the day may influence the total daily energy intake.

O’Neil et al. proposed that a range between 15% and 25% energy contribution should be covered at breakfast [30]. We observed that the association between the percentage of energy from breakfast and the prevalence of MetS was below the unit whenever the percentage of energy covered at breakfast ranged between 15 and 29% of the total daily energy intake. These results are therefore in agreement with those by O’Neil’s.

Whereas the mechanisms involved in the effects of the breakfast size on metabolism and weight-related health remain unclear, they may include both behavioral patterns–such as increased or decreased physical activity, and food choice– and the influence of circadian rhythms on energy metabolism [8,44]. As for the latter, diet induced thermogenesis has been proven to be under circadian control, with levels in the morning being higher than later in the day [8]. Experimental designs have reinforced that consuming greater amounts of energy earlier, rather than later in the day, enhances the metabolic response regardless of the meal’s nutrient composition [25,45,46,47]. Increased diet-induced thermogenesis can, in turn, enhance overall energy expenditure and improve the glycemic response, both resulting in better management of cardiometabolic health [45]. Furthermore, it has also been shown that intake of a high-energy-density diet in the morning–regardless of the food consumed–increases satiety and can reduce the total ingested amount for the rest of the day [31,48]. 

We would like to mention that, before our study, available evidence raised from small samples, which implies very heterogenous populations and, consequently, make their results not suitable for comparison; breakfast patterns could vary across countries and age of the participants [30]. Furthermore, most of them were based on a single 24-h recall, so that results must be interpreted with caution, as they may not be indicative of the usual breakfast intakes. A consensus on definitions of breakfast, time of breakfast, and breakfast size is still required.

The main limitation of our study was that our data were obtained cross-sectionally, and so it does not allow for a determination of causality. Self-reported bias was likely for sleep duration, as well as for the individual’s chronotype despite validated questionnaires being used for the assessment of these variables. Information on medication for hypertriglyceridemia, HDL-cholesterol, hypertension, and hyperglycemia was not collected, so prevalence of MetS could be underestimated. We would like to highlight that the present study involved a large sample from five Spanish regions and included the use of diet history questionnaires, structured by eating occasion, which enabled us to collect detailed information on individual food intake, meal timing and to estimate macronutrients and breakfast size. 

In conclusion, our results show that high breakfast size is associated to total daily energy intake and may decrease the prevalence of MetS, as observed in a large sample of Spanish older adults. Our findings reinforce the importance of breakfast size as a potential strategy to reduce the prevalence of MetS, since in modern societies adequate meal timing and energy distribution during the day are difficult to achieve. Further research in prospective studies or randomized control trial in the general adult population are needed to confirm the role, if any, of breakfast size in relation to MetS.

## Figures and Tables

**Table 1:** Descriptive characteristics by Metabolic Syndrome Status.

	Metabolic Syndrome (MetS) ^1^		
	No (*n* = 2160)	Yes (*n* = 1484)	*p* Value ^2^
Number of Diagnostic Criteria			
0	70		
1	888		
2	1202		
3		989	
4		395	
5		100	
Presence of individual diagnostic of MetS			
Waist circumference, yes	510 (28.1)	1305 (71.9)	<0.001
Triglycerides, yes	87 (13.1)	579 (86.9)	<0.001
HDL-Cholesterol, yes	36 (9.1)	359 (90.9)	<0.001
Hypertension, yes	1973 (57.2)	1476 (42.8)	<0.001
Hyperglycemia, yes	686 (34.1)	1328 (65.9)	<0.001
Study center			
Asturias	538 (61.1)	343 (38.9)	<0.001
Granada	436 (59.8)	293 (40.2)	
Murcia	370 (46.8)	421 (53.2)	
Navarra	359 (59.7)	242 (40.3)	
Guipuzkoa	457 (71.2)	185 (28.8)	
Sex			
Men	880 (60.7)	569 (39.3)	0.146
Women	1280 (58.3)	915 (41.7)	
Age (years)	65.00 (61.68–68.54)	65.94 (62.12–69.11)	<0.001
Educational level			
None	327 (48.7)	344 (51.3)	<0.001
Primary school	737 (55.9)	581 (44.1)	
Technical studies	310 (62.9)	183 (37.1)	
Secondary school	267 (61.5)	167 (38.5)	
Higher education	513 (72.0)	200 (28.1)	
Missing	6 (40.0)	9 (60.0)	
Smoking history			
Never	1080 (59.4)	737 (40.6)	0.872
Current	259 (57.6)	191 (42.4)	
Former	819 (59.6)	555 (40.4)	
Missing	1 (50.0)	1 (50.0)	
Body Mass Index (kg/m^2^)	26.29 (24.10–28.60)	30.80 (28.30–33.70)	<0.001
% Body fat	29.15 (23.60–35.15)	35.50 (29.60–40.10)	<0.001
Non-laboral physical activity (MET-h/week)	80.00 (58.75–104.30)	77.55 (53.50–102.25)	0.007
At home	39.90 (21.00–62.30)	44.05 (23.10–66.48)	<0.001
Recreation	36.00 (22.50–51.00)	30.00 (18.00–44.38)	<0.001
Sleep quality			
Good	2109 (59.6)	1430 (40.4)	0.024
Poor	51 (48.6)	54 (51.4)	
Time of sleep (h/workdays)	6:25 (7:10–7:55)	6:25 (7:15–7:55)	0.475
Time of sleep (h/freedays)	6:35 (7:29–8:10)	6:30 (7:25–8:15)	0.503
Chronotype			
Moderate/extreme early type	54 (57.5)	40 (42.6)	0.245
Slight early type	151 (59.2)	104 (40.8)	
Normal type	1717 (60.0)	1147 (40.1)	
Slight late type	155 (58.5)	110 (41.5)	
Moderate/extreme late type	45 (49.5)	46 (50.6)	
Missing	38 (50.7)	37 (49.3)	
Breakfast time (h)			
≤9:00	1686 (62.1)	1028 (37.9)	<0.001
>9:00	468 (51.2)	446 (48.8)	
Missing	6 (37.5)	10 (62.5)	
Lunch time (h)			
≤14:00	1041 (60.4)	680 (39.6)	0.039
>14:00–15:00	774 (57.1)	580 (42.9)	
>15:00	165 (65.5)	87 (34.5)	
Missing	180 (56.8)	137 (43.2)	
Dinner time (h)			
≤21:00	677 (58.6)	477 (41.3)	0.364
21:00	577 (60.7)	372 (39.2)	
>21:00	667 (59.8)	447 (40.2)	
Missing	239 (56.0)	188 (44.0)	
Number of eating occasions			0.004
≤3	93 (64.6)	51 (35.4)	
4–5	990 (61.8)	612 (38.2)	
>5	1077 (56.7)	821 (43.3)	
Breakfast size (×2000 kcal) ^3^	306.85 (229.81–391.63)	280.42 (205.36–368.18)	<0.001
Dietary intake at breakfast			
Energy at breakfast from the total (%)	15.34 (11.49–19.58)	14.02 (10.27–18.41)	<0.001
Macronutrient composition (%)			
Proteins	13.73 (11.17–16.96)	14.28 (11.46–17.51)	0.005
Carbohydrates	56.59 (48.84–64.70)	55.09 (47.58–62.81)	<0.001
Fats	28.26 (18.97–37.16)	29.49 (19.98–38.01)	0.059
Total energy at entire day (kcal/day)	2386.2 (2140.9–2678.9)	2489.3 (2218.0–2788.6)	<0.001

Data are expressed in n (MetS prevalence) and median (p25-p75). ^1^ Metabolic Syndrome (MetS) was defined according to the National Cholesterol Education Program’s Adult Treatment Panel III (ATP III) definition, when an individual had three or more of the following diagnostic criteria: waist circumference ≥ 102 cm in men and ≥88 cm in women; triglycerides ≥ 150 mg/dL (1.695 mmol/L); HDL-cholesterol < 40 mg/dL (0.9 mmol/L) in men and <50 mg/dL (1.1 mmol/L) in women; blood pressure ≥ 130/85 mmHg; and fasting glucose ≥ 100 mg/dL (≥6.1 mmol/L). ^2^ *p* value: Chi square test for categorical variables and Wilcoxon rank test for continuous variables. ^3^ Breakfast size was calculated as (energy from breakfast/total energy intake) * 2000 kcal.

**Table 2:** Descriptive characteristics by quartiles of breakfast size (×2000 kcal).

	Breakfast Size (×2000 kcal) ^1^				
	Q1 (<222) (*n* =911)	Q2 (222–296.3) (*n* = 911)	Q3 (296.4–384) (*n* = 911)	Q4 (>384) (*n* = 911)	*p* Value ^2^
Number of diagnostic criteria					
0	9 (1.0)	19 (2.1)	14 (1.5)	28 (3.1)	<0.001
1	157 (17.2)	212 (23.3)	247 (27.1)	272 (29.9)	
2	308 (33.8)	303 (33.3)	300 (32.9)	291 (31.9)	
3	286 (31.4)	238 (26.1)	247 (27.1)	218 (23.9)	
4	118 (13.0)	109 (12.0)	80 (8.8)	88 (9.7)	
5	33 (3.6)	30 (3.3)	23 (2.5)	14 (1.5)	
MetS ^3^, yes	437 (48.0)	377 (41.4)	350 (38.4)	320 (35.1)	<0.001
Presence of individual components of MetS ^3^					
Abdominal obesity, yes	509 (55.9)	473 (51.9)	439 (48.2)	394 (43.3)	<0.001
Triglycerides, yes	195 (21.4)	170 (18.7)	149 (16.4)	152 (16.7)	0.020
HDL-Cholesterol, yes	113 (12.4)	103 (11.3)	87 (9.6)	92 (10.1)	0.204
Hypertension, yes	865 (95.0)	859 (94.3)	871 (95.6)	854 (93.7)	0.317
Hyperglycemia, yes	586 (64.3)	513 (56.3)	477 (52.4)	438 (48.1)	<0.001
Study center					
Asturias	245 (26.9)	222 (24.4)	215 (23.6)	199 (21.8)	<0.001
Granada	116 (12.7)	170 (18.7)	233 (25.6)	210 (23.1)	
Murcia	194 (21.3)	231 (25.4)	183 (20.1)	183 (20.1)	
Navarra	190 (20.9)	138 (15.2)	128 (14.1)	145 (15.9)	
Guipúzcoa	166 (18.2)	150 (16.5)	152 (16.7)	174 (19.1)	
Sex					
Men	429 (47.1)	377 (41.4)	321 (35.2)	322 (35.4)	<0.001
Women	482 (52.9)	534 (58.6)	590 (64.8)	589 (64.7)	
Age (year)	65.25 (61.91–68.62)	65.31 (62.00–68.53)	65.09 (61.82–68.47)	65.92 (62.00–69.54)	0.044
Educational level					
None	154 (16.9)	174 (19.1)	179 (19.7)	164 (18.0)	<0.001
Primary school	367 (40.3)	367 (40.3)	279 (30.6)	305 (33.5)	
Technical studies	115 (12.6)	122 (13.4)	133 (14.6)	123 (13.5)	
Secondary school	122 (13.4)	105 (11.5)	110 (12.1)	97 (10.7)	
Higher education	150 (16.5)	142 (15.6)	206 (22.6)	215 (23.6)	
Missing	3 (0.3)	1 (0.1)	4 (0.4)	7 (0.8)	
Smoking history					
Never	369 (40.5)	472 (51.8)	486 (53.4)	490 (53.9)	<0.001
Current	169 (18.6)	102 (11.2)	95 (10.4)	84 (9.2)	
Former	373 (40.9)	337 (37.0)	329 (36.1)	335 (36.8)	
Missing			1 (0.1)	1 (0.1)	
Body Mass Index (kg/m^2^)	28.82 (26.10–31.90)	28.20 (25.60–31.30)	27.84 (25.40–30.70)	26.90 (24.50–30.30)	<0.001
% Body fat	32.00 (25.90–37.70)	31.70 (25.70–37.30)	32.40 (25.60–37.70)	31.80 (25.90–36.80)	0.543
Non-laboral Physical activity (MET-h/week)	76.60 (53.80–100.80)	79.80 (55.20–105.00)	77.80 (56.20–102.30)	81.30 (61.10–104.85)	0.023
At home	41.20 (20.80–62.30)	42.70 (20.30–65.80)	43.20 (23.60–65.60)	42.00 (22.60–63.53)	0.456
Recreational	31.50 (19.50–48.00)	33.00 (21.00–48.00)	31.50 (21.00–48.00)	36.00 (21.00–51.00)	0.0002
Sleep quality					
Good	878 (96.4)	884 (97.0)	895 (98.2)	882 (96.8)	0.101
Poor	33 (3.6)	27 (3.0)	16 (1.8)	29 (3.2)	
Time of sleep (h/workdays)	6:25 (7:15–7:55)	6:25 (7:05–7:55)	6:30 (7:10–7:55)	6:25 (7:10–7:55)	0.748
Time of sleep (h/freedays)	6:30 (7:25–8:10)	6:30 (7:25–8:10)	6:45 (7:30–8:10)	6:30 (7:28–8:10)	0.283
Chronotype					
Moderate/extreme early type	23 (2.5)	21 (2.3)	23 (2.5)	27 (3.0)	0.003
Slight early type	54 (5.9)	58 (6.4)	71 (7.8)	72 (7.9)	
Normal type	696 (76.4)	731 (80.2)	710 (77.9)	727 (79.8)	
Slight late type	82 (9.0)	58 (6.4)	72 (7.9)	53 (5.8)	
Moderate/extreme late type	34 (3.7)	25 (2.7)	24 (2.6)	8 (0.9)	
Missing	22 (2.4)	18 (2.0)	11 (1.2)	24 (2.6)	
Breakfast time (h)					
≤9:00	690 (75.7)	683 (75.0)	653 (71.7)	688 (75.5)	0.075
>9:00	207 (22.7)	227 (24.9)	257 (28.2)	223 (24.5)	
Missing	14 (1.5)	1 (0.1)	1 (0.1)		
Lunch time (h)					
≤14:00	450 (49.4)	415 (45.6)	416 (45.7)	440 (48.3)	0.114
>14:00–15:00	304 (33.4)	349 (38.3)	361 (39.6)	340 (37.3)	
>15:00	60 (6.6)	66 (7.2)	63 (6.9)	63 (6.9)	
Missing	97 (10.7)	81 (8.9)	71 (7.8)	68 (7.5)	
Dinner time (h)					
≤21:00	295 (32.4)	295 (32.4)	271 (29.8)	293 (32.2)	0.028
21:00	222 (24.4)	221 (24.3)	229 (25.1)	277 (30.4)	
>21:00	286 (31.4)	279 (30.6)	297 (32.6)	252 (27.7)	
Missing	108 (11.9)	116 (12.7)	114 (12.5)	89 (9.8)	
Number of eating occasions					0.0001
≤3	32 (3.5)	32 (3.5)	25 (2.7)	55 (6.0)	
4–5	385 (42.3)	373 (40.9)	413 (45.3)	431 (47.3)	
>5	494 (54.2)	506 (55.5)	473 (51.9)	425 (46.7)	
Dietary intake at breakfast					
Energy at breakfast from the total (%)	7.80 (5.07–9.73)	13.05 (12.08–13.91)	16.80 (15.72–17.88)	22.83 (20.57–26.31)	
Macronutrient composition (%)					
Proteins	16.08 (11.73–20.65)	14.63 (12.23–17.20)	13.66 (11.63–16.17)	12.40 (10.24–15.04)	<0.001
Carbohydrates	58.33 (50.29–68.01)	56.78 (49.60–64.29)	55.63 (48.07–63.55)	53.36 (44.44–60.45)	<0.001
Fats	23.03 (9.95–31.87)	27.83 (19.38–35.87)	29.57 (21.02–38.39)	33.38 (25.32–42.37)	<0.001
Total energy intake (kcal/day)	2494.1 (2239.2–2847.7)	2458.6 (2204.7–2749.2)	2396.2 (2155.7–2685.2)	2322.3 (2070.8–2626.3)	<0.001

Data are expressed in n (%) and median (p25-p75). ^1^ Breakfast size was calculated as (energy from breakfast/total energy intake) * 2000 kcal. ^2^ *p* value: Chi square test for categorical variables and Wilcoxon rank test for continuous variables. ^3^ Metabolic Syndrome (MetS) was defined according to the National Cholesterol Education Program’s Adult Treatment Panel III (ATP III) definition, when an individual had three or more of the following diagnostic criteria: waist circumference ≥ 102 cm in men and ≥88 cm in women; triglycerides ≥ 150 mg/dL (1.695 mmol/L); HDL-cholesterol < 40 mg/dL (0.9 mmol/L) in men and <50 mg/dL (1.1 mmol/L) in women; blood pressure ≥ 130/85 mmHg; and fasting glucose ≥ 100 mg/dL (≥6.1 mmol/L).

**Table 3:** Association (OR, 95% CI) between energy contribution from breakfast and prevalence of Metabolic Syndrome ^1^ and for each individual component.

	Breakfast Size ^2^			
	Q1 (<222)	Q2 (222–296.3)	Q3 (296.4–384)	Q4 (>384)
Prevalence of MetS				
Model 1	1.00 (referent)	0.72 (0.60–0.88)	0.65 (0.53–0.78)	0.55 (0.45–0.67)
Model 2	1.00 (referent)	0.74 (0.61–0.90)	0.70 (0.56–0.83)	0.62 (0.51–0.76)
Presence of individual components of MetS				
Abdominal obesity ^3^	1.00 (referent)	0.78 (0.64–0.95)	0.68 (0.56–0.83)	0.59 (0.48–0.72)
Triglycerides ^3^	1.00 (referent)	0.88 (0.70–1.11)	0.75 (0.59–0.96)	0.84 (0.66–1.07)
HDL-Cholesterol ^3^	1.00 (referent)	0.92 (0.57–1.04)	0.77 (0.57–1.04)	0.87 (0.65–1.18)
Hypertension ^3^	1.00 (referent)	0.75 (0.49–1.16)	0.83 (0.53–1.32)	0.57 (0.37–0.88)
Hyperglycemia ^3^	1.00 (referent)	0.73 (0.60–0.89)	0.67 (0.54–0.81)	0.56 (0.46–0.68)

Model 1: adjusted for center, sex, age. Model 2: adjusted for center, sex, age, educational level, recreational physical activity, number of eating occasions (≤3, 4–5, >5), and breakfast time (≤9:00, >9:00). ^1^ Metabolic Syndrome (MetS) was defined according to the National Cholesterol Education Program’s Adult Treatment Panel III (ATP III) definition, when an individual had three or more of the following diagnostic criteria: waist circumference ≥ 102 cm in men and ≥ 88 cm in women; triglycerides ≥ 150 mg/dL (1.695 mmol/L); HDL-cholesterol < 40 mg/dL (0.9 mmol/L) in men and <50 mg/dL (1.1 mmol/L) in women; blood pressure ≥ 130/85 mmHg; and fasting glucose ≥ 100 mg/dL (≥6.1 mmol/L). ^2^ Breakfast size was calculated as (energy from breakfast/total energy intake) * 2000 kcal. ^3^ Model adjusted for center, sex, age, educational level, recreational physical activity, number of eating occasions (≤3, 4–5, >5), and breakfast time (≤9:00, >9:00).

## Supplementary Materials

The following supporting information can be downloaded at: https://www.mdpi.com/article/10.3390/nu15030630/s1: Table S1: Descriptive dietary information by Metabolic Syndrome. Table S2: Descriptive dietary information by quartiles of breakfast size (×2000 kcal). Table S3: Odds ratio (95% CI) for chrono-nutrition variables included in the logistic regression model for Metabolic Syndrome. Figure S1. Flowchart Data collection.
