# Supplementary material for: Breakfast Size and Prevalence of Metabolic Syndrome in the European Prospective Investigation into Cancer and Nutrition (EPIC) Spanish Cohort
Source: Nutrients. 2023 Jan 26;15(3):630. doi: 10.3390/nu15030630 (PMC9919450; doi:10.3390/nu15030630)
Supplement: Supplementary file 1 [file nutrients-15-00630-s001.zip › nutrients-2096804-supplementary.pdf]

Supplemental Table S1. Descriptive dietary information by Metabolic Syndrome

	Metabolic syndrome (MetS)[1]		
	**No (n = 2,160)**	**Yes (n = 1,484)**	***p* value[2]**
**Dietary intake at breakfast**			
Macronutrients (g/breakfast)			
Total carbohydrates	50.39 (36.86- 66.88)	46.36 (33.81- 61.68)	<0.001
Simple carbohydrates	24.83 (16.37- 34.93)	21.01 (13.87- 30.36)	<0.001
Fibre	3.38 (1.96- 5.55)	2.86 (1.66- 5.07)	<0.001
Total proteins	12.62 (9.19- 16.20)	12.25 (9.12- 16.01)	0.196
Animal protein	6.60 (3.30- 9.28)	6.60 (4.00- 9.57)	0.204
Plant-based protein	6.02 (3.75- 8.10)	5.75 (3.30- 7.59)	0.001
Fats	10.94 (6.06- 17.46)	10.62 (6.07- 16.59)	0.455
Saturated fats	3.20 (1.64- 5.43)	3.26 (1.76- 5.55)	0.541
Monounsaturated fats	4.25 (1.88- 7.27)	4.24 (1.81- 7.41)	0.964
Polyunsaturated fats	1.25 (0.69- 2.41)	1.21 (0.64- 2.22)	0.048
**Dietary intake at full day**			
Macronutrient composition (%)			
Proteins	18.31 (16.90- 19.95)	18.46 (17.01- 20.16)	0.057
Carbohydrates	41.60 (37.91- 45.53)	41.41 (37.75- 45.08)	0.102
Fats	36.91 (33.25- 40.53)	36.80 (33.52- 40.26)	0.850
Macronutrients (g/day)			
Total carbohydrates	248.84 (218.59-282.88)	257.25 (225.79-291.05)	<0.001
Simple carbohydrates	94.51 (77.51-112.45)	90.30 (74.30-109.13)	<0.001
Fibre	36.29 (30.31- 43.83)	37.15 (30.54- 43.88)	0.244
Total proteins	110.12 (96.63-124.71)	115.19 (102.24-131.02)	<0.001
Animal proteins	66.26 (54.81- 79.41)	70.23 (59.16- 83.41)	<0.001
Plant-based proteins	43.01 (36.88- 49.92)	44.76 (38.18- 52.01)	<0.001
Fats	97.61 (83.00-114.85)	101.47 (86.73-119.05)	<0.001
Saturated fats	25.61 (20.86- 31.88)	27.14 (22.04- 33.56)	<0.001
Monounsaturated fats	44.25 (36.67- 52.77)	46.00 (38.59- 54.11)	<0.001
Polyunsaturated fats	17.17 (13.54- 22.42)	17.65 (13.86- 22.51)	0.158

Data are expressed in median (p25-p75).

[1]Metabolic Syndrome (MetS) was defined according to the National Cholesterol Education Program's Adult Treatment Panel III (ATP III) definition. It was considered present (Yes) when an individual fulfilled three or more of the following diagnostic criteria: waist circumference ≥102 cm in men and ≥88 cm in women; triglycerides ≥150 mg/dl (1.695 mmol/l); HDL-cholesterol <40 mg/dl (0.9 mmol/l) in men and <50 mg/dl (1.1 mmol/l) in women; blood pressure ≥130/85 mmHg; and fasting glucose ≥100 mg/dl (≥6.1 mmol/l).

[2]p value: Wilcoxon rank test

[3] Breakfast size was calculated as: (energy from breakfast/total energy intake)*2,000kcal

Supplemental Table S2. Descriptive dietary information by quartiles of breakfast size (x2000kcal)

	Breakfast size (*2,000 kcal)[1]				
	Q1 (<222) (n=911)	Q2 (222-296.3) (n=911)	Q3 (296.4-384) (n=911)	Q4 (>384) (n=911)	p value[2]
**Dietary intake at breakfast**					
Macronutrient intake (g/breakfast)					
Total carbohydrates	27.64 (17.52- 36.63)	44.82 (37.75- 53.48)	56.13 (46.90- 66.63)	72.11 (57.76- 87.90)	<0.001
Simple carbohydrates	15.69 (9.83- 22.26)	21.39 (14.94- 28.44)	26.68 (18.52- 35.52)	32.88 (22.83- 45.71)	<0.001
Fibre	1.37 (0.20- 2.85)	2.73 (1.82- 4.53)	3.71 (2.45- 5.55)	5.30 (3.47- 7.82)	<0.001
Total proteins	7.39 (4.89- 10.10)	11.71 (9.65- 13.77)	13.83 (11.63- 16.51)	17.07 (13.68- 21.21)	<0.001
Animal proteins	4.96 (1.64- 6.60)	6.60 (4.14- 8.28)	6.64 (5.03- 9.67)	7.21 (4.81- 10.56)	<0.001
Plant-based proteins	2.24 (0.92- 4.21)	5.24 (3.92- 6.60)	6.53 (5.15- 8.10)	8.92 (6.69- 12.46)	<0.001
Fats	4.47 (1.69- 7.50)	10.04 (6.66- 13.31)	13.02 (9.08- 17.86)	20.49 (14.10- 28.03)	<0.001
Saturated fats	1.60 (0.34- 3.03)	2.97 (1.72- 4.62)	3.75 (2.19- 5.74)	5.46 (3.22- 8.20)	<0.001
Monounsaturated fats	1.40 (0.35- 2.70)	3.87 (2.17- 5.90)	5.31 (3.27- 7.75)	8.24 (5.26- 12.53)	<0.001
Polyunsaturated fats	0.47 (0.14- 0.83)	1.11 (0.73- 1.54)	1.49 (1.01- 2.62)	2.64 (1.50- 5.45)	<0.001
**Dietary intake at full day**					
Macronutrient composition (%)					
Proteins	18.82 (17.28- 20.48)	18.54 (17.12- 20.26)	18.28 (17.03- 19.85)	17.92 (16.38- 19.53)	<0.001
Carbohydrates	39.73 (35.70- 43.59)	41.44 (38.30- 44.93)	41.94 (38.41- 45.72)	42.68 (39.00- 46.72)	<0.001
Fats	37.01 (33.45- 41.09)	36.72 (33.76- 39.73)	36.78 (33.20- 40.44)	36.91 (33.17- 40.70)	0.220
Macronutrient intake (g/day)					
Total carbohydrates	250.35(218.71-283.94)	254.56(225.51-287.14)	254.01(223.92-287.09)	248.61(215.52-287.17)	0,034
Simple carbohydrates	86.37 (70.48-104.79)	91.73 (75.21-109.47)	94.11 (78.65-112.47)	98.92 (80.60-118.24)	<0.001
Fibre	37.35 (30.49- 45.44)	38.29 (31.85- 44.78)	36.27 (30.47- 43.21)	35.21 (29.12- 42.29)	<0.001
Total proteins	118.56(104.07-133.83)	115.56(101.11-130.78)	111.11 (98.59-124.08)	104.90 (92.41-118.98)	<0.001
Animal proteins	73.49 (61.32- 87.66)	69.44 (58.27- 83.36)	66.90 (56.71- 78.34)	62.27 (50.27- 74.21)	<0.001
Plant-based proteins	44.65 (38.29- 52.04)	45.04 (38.75- 51.46)	43.16 (36.95- 50.04)	41.83 (35.92- 49.10)	<0.001

Fats	103.86 (86.90-122.19)	100.06 (86.00-115.07)	98.20 (84.29-115.17)	96.67 (81.01-113.69)	<0.001
Saturated fats	27.71 (22.51- 35.12)	26.57 (21.82- 32.64)	26.26 (21.06- 32.02)	24.95 (20.08- 30.94)	<0.001
Monounsaturated fats	46.52 (38.66- 55.33)	45.19 (38.49- 53.40)	44.80 (37.82- 52.75)	43.08 (35.04- 52.46)	<0.001
Polyunsaturated fats	17.97 (14.01- 23.49)	17.29 (14.08- 21.98)	16.77 (13.44- 22.01)	17.45 (13.15- 22.37)	0.002

Data are expressed in median (p25-p75).

[1] Breakfast size was calculated as: (energy from breakfast/total energy intake)*2000kcal

[2] p value: Wilcoxon rank test

Supplemental Table S3. Odds ratio (95%CI) for chrono-nutrition variables included in the logistic regression model for Metabolic Syndrome.

**Breakfast time (h)**		
	≤9:00	1.00 (referent)
	>9:00	1.34 (1.14-1.57)
**Number of eating occasions**		
	≤3	0.80 (0.55-1.17)
	4-5	1.00 (referent)
	>5	1.23 (1.06-1.42)

Model: adjusted for centre, sex, age, educational level, recreational physical activity, number of eating occasions (≤3, 4-5, >5), breakfast time (≤9:00, >9:00 h), and breakfast size

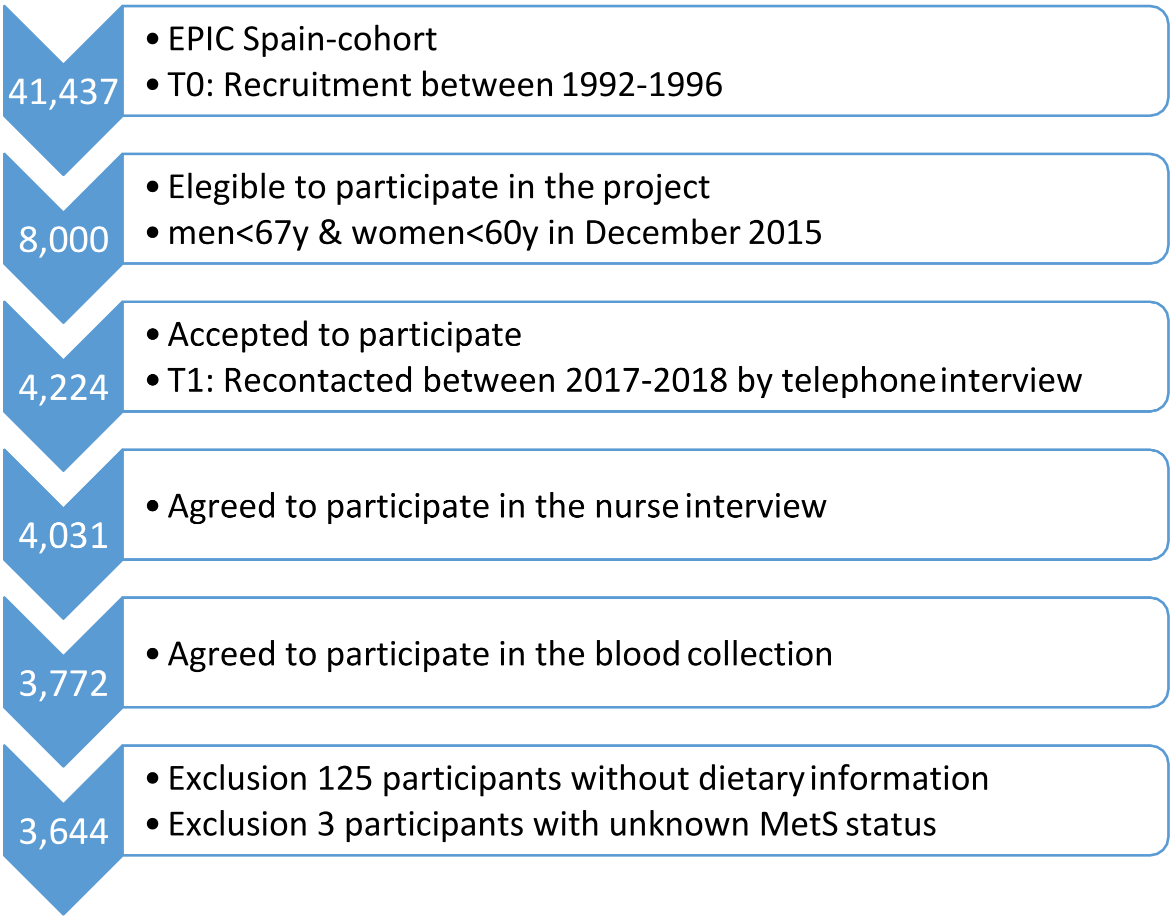

Supplemental Figure S1. Flowchart Data collection
